# A transcriptome analysis focusing on inflammation-related genes of grass carp intestines following infection with *Aeromonas hydrophila*

**DOI:** 10.1038/srep40777

**Published:** 2017-01-17

**Authors:** Xuehong Song, Xiaolong Hu, Bingyao Sun, Yunxuan Bo, Kang Wu, Lanying Xiao, Chengliang Gong

**Affiliations:** 1 School of Biology and Basic Medical Sciences, Soochow University, Suzhou 215123, China; 2 National Engineering Laboratory for Modern Silk, Soochow University, Suzhou 215123, China

## Abstract

Inflammation is a protective response that is implicated in bacterial enteritis and other fish diseases. The inflammatory mechanisms behind *Aeromonas hydrophila* infections in fish remain poorly understood. In this study, we performed a *de novo* grass carp transcriptome assembly using Illumina’s Solexa sequencing technique. On this basis we carried out a comparative analysis of intestinal transcriptomes from *A. hydrophila*-challenged and physiological saline solution (PSS/mock) -challenged fish, and 315 genes were up-regulated and 234 were down-regulated in the intestines infected with *A. hydrophila*. The GO enrichment analysis indicated that the differentially expressed genes were enriched to 12, 4, and 8 GO terms in biological process, molecular function, and cellular component, respectively. A KEGG analysis showed that 549 DEGs were involved in 165 pathways. Moreover, 15 DEGs were selected for quantitative real-time PCR analysis to validate the RNA-seq data. The results confirmed the consistency of the expression levels between RNA-seq and qPCR data. In addition, a time-course analysis of the mRNA expression of 12 inflammatory genes further demonstrated that the intestinal inflammatory responses to *A. hydrophila* infection simultaneously modulated gene expression variations. The present study provides intestine-specific transcriptome data, allowing us to unravel the mechanisms of intestinal inflammation triggered by bacterial pathogens.

The grass carp (*Ctenopharyngodon idella*) is an intensively cultured and economically important herbivorous fish species in China. In grass carp aquaculture, the fish are often infected by bacterial, viral and parasitic pathogens. *Aeromonas hydrophila*, a Gram-negative aquatic bacterium, is regarded as the major bacterial pathogen causing intestinal inflammation in animal taxa, including farmed fish^1^,^2^,^3^,^4^. *A. hydrophila*-induced enteritis is probably the most widespread disease in grass carp, especially under intensive rearing conditions, which results in huge economic losses annually due to reduced growth and high mortality rates^5^,^6^,^7^. The molecular mechanisms and pathways that regulate bacterial-induced inflammatory processes in humans and other mammalian species are relatively well understood^8^,^9^,^10^. In fish, several inflammation-related genes, including chemokine (C-X-C motif) ligand 2 (CXCL2)^11^, CXCL10^12^, cyclooxygenase-2 (COX-2)^13^, interleukin-1β (IL-1β)^4^,^13^,^14^, IL-8^15^,^16^, IL-17A/F2^17^, and tumor necrosis factor (TNF)-α^13^,^18^, have already been identified and characterized with regard to their involvement in inflammatory responses to bacterial infections. However, the definitive mechanisms responsible for the bacterial-induced intestinal inflammation in fish are still unclear. Therefore, further studies are required to identify more molecular components that mediate inflammatory reactions in fish.

In the past few years, transcriptome sequencing technology has found broad applications in gene discovery and gene expression profiling. Recently, a number of studies have generated abundant transcriptome data from blue catfish (*Ictalurus furcatus*)^19^, blunt snout bream (*Megalobrama amblycephala*)^20^, grass carp^21^,^22^, and rohu (*Labeo rohita*)^23^ after being infected with *A. hydrophila*. These studies have primarily focused on identifying immune-related genes and substantially improved our understanding of the host immune defense mechanisms against bacterial infection. However, no special emphasis was given to systematically discover inflammation-related genes using a transcriptome analysis, although several pathogenic bacteria have been reported to cause severe inflammation in various farmed fish species^5^,^7^,^24^,^25^,^26^,^27^. In fact, inflammation is a normal biological response of the host immune system to noxious stimuli and conditions, such as bacterial infection and tissue injury, which allows for the repair of damaged or infected tissue. In intensive aquaculture, severe and widespread inflammation can develop more readily in the intestines than in other tissues, largely due to a heavier pathogenic bacteria load in the intestinal lumen or a more effective colonization of the intestinal mucosa^28^,^29^,^30^.

Recently, pathological studies have consistently indicated that *A. hydrophila* causes various intestinal and extra-intestinal diseases, from relatively mild enteritis (gastroenteritis in humans)^5^,^27^,^31^,^32^,^33^ to potentially fatal septicemia^32^,^33^,^34^,^35^, in humans and other mammals, reptiles, birds, and fish species^27^,^31^,^32^,^33^,^34^,^35^. In grass carp, septicemia has received more attention than *A. hydrophila*-induced enteritis because miRNAs implicated in motile aeromonad septicemia were identified by deep sequencing^36^,^37^.

To date, only a few intestine-specific fish transcriptomes have been reported and none have been described in the context of bacteria-induced intestinal inflammation. Thus, this study aimed to identify novel candidate genes that are functionally implicated in intestinal inflammation. To do this, a grass carp transcriptome from seven different tissues was sequenced, *de novo* assembled, and used to compare transcriptome profiles of *A. hydrophila*-infected or physiological saline solution (PSS/mock)-infected grass carp intestinal samples. The transcriptome sequence data generated in this study will be powerful aids to enhance our understanding of the pathogenic mechanisms of bacterial enteritis in grass carp and other farmed fish.

## Results

### Illumina sequencing and *de novo* assembly

A cDNA library of seven different tissues (thymus, head kidney, spleen, liver, skin, gill, and intestine) from healthy grass carp was constructed by Illumina sequencing in a single run. After removing the low-quality reads, a total of 45,867,282 clean reads and 4,128,055,380 clean nucleotides were obtained, and assembled into 120,964 contigs, with an average length of 369 bp, and 67,413 unigenes (11,623 clusters and 55,790 singletons), with an average length of 665 bp (Supplementary Tables S1 and S2, and Supplementary Fig. S1). All of the non-redundant transcripts were treated as a transcriptome database to identify genes associated with intestinal inflammation using DGEs. The transcriptome sequencing data generated in this study have been deposited in NCBI BioSample database under accession number SAMN04569528.

### Annotation of predicted proteins

**Figure 1:**
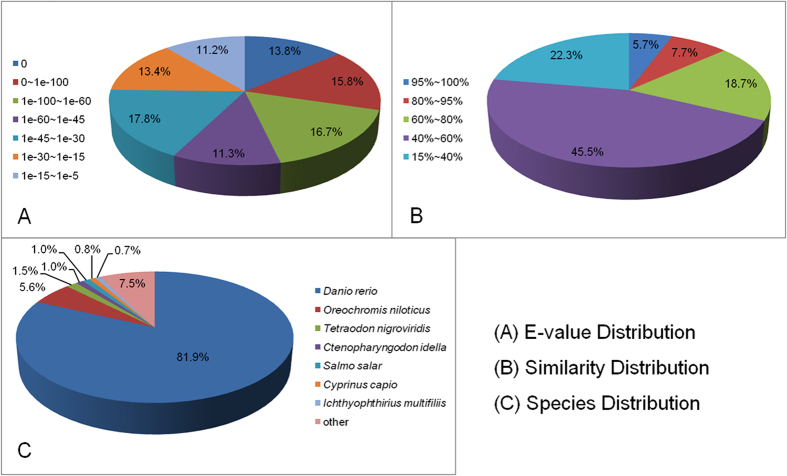
BLASTX analysis of unigenes identified from the grass carp transcriptome. A total of 57,532 unigenes were queried against the nonredundant (nr) protein database. The pie charts show the E-value distribution (**A**), similarity distribution (**B**) and species distribution (**C**) of the BLASTX matches to the unigenes.

Unigene sequences were annotated by searching the nr NCBI protein database using the BLASTX algorithm, with a cut-off E-value of 10^−5^. A total of 57,532 distinct sequences (82.76%) of unigenes matched known genes (Fig. 1). The E-value distribution indicated that 57.6% of the mapped unigenes were significantly homologous, with E-values less than 1e^−45^, while the remaining 42.4% had E-values ranging from 1e^−45^ to 1e^−5^ (Fig. 1A). In addition, a similarity distribution analysis showed that 67.8% of unigenes share more than 80.0% similarity with available reference sequences (Fig. 1B). The majority (82.0%) of the sequences had a strong homology with those of *Danio rerio*, followed by those of *Oreochromis niloticus* (5.6%), *Tetraodon nigroviridis* (1.5%), *C. idella* (1.0%), *Salmo salar* (1.0%), *Cyprinus carpio* (0.7%), *Ichthyophthirius multifiliis* (0.7%), and other species (7.5%) (Fig. 1C).

### Functional annotations of the unigenes

**Figure 2:**
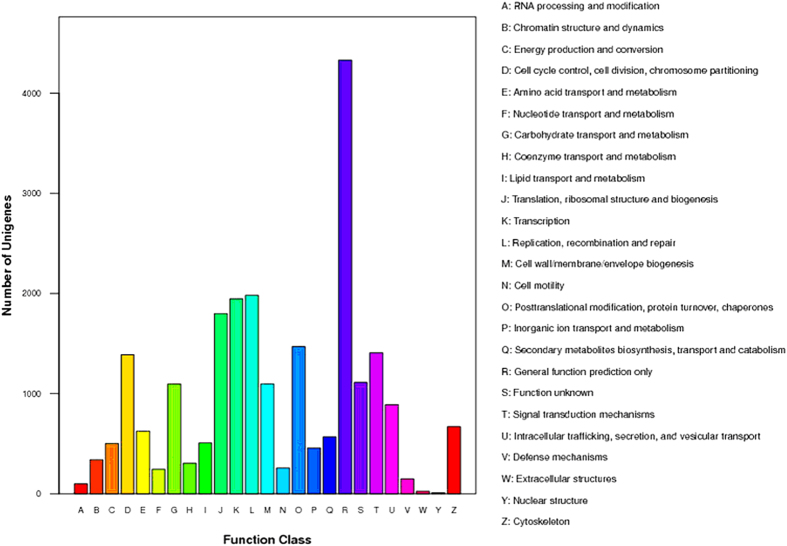
Clusters of orthologous groups (COGs) functional classification of unigenes identified from the grass carp transcriptome. A total of 23,275 unigenes were annotated and divided into 25 specific categories.

**Figure 3:**
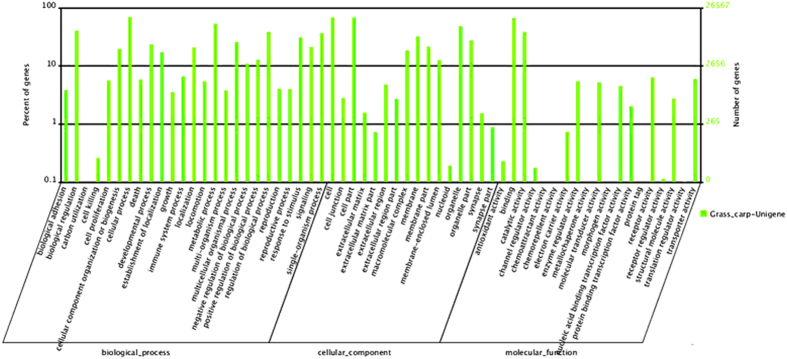
Gene ontology (GO) classifications of unigenes identified from the grass carp transcriptome. The unigenes were classified into 60 subcategories under the three main GO categories: biological process, cellular component and molecular function.

The COG database was used to predict the possible functions of the unigenes. In total, 23,275 unigenes were annotated and divided into 25 categories (Fig. 2). Among these categories, the general function prediction cluster, representing the largest group, contained 4330 unigenes (18.6%), followed by replication, recombination and repair (1982 unigenes, 8.5%), transcription (1947 unigenes, 8.4%), translation, ribosomal structure and biogenesis (1800 unigenes, 7.7%), post-translational modification, protein turnover, chaperones (1471 unigenes, 6.3%), signal transduction mechanisms (1408 unigenes, 6.0%), cell cycle control, cell division, chromosome partitioning (1388 unigenes, 5.96%), and others (Fig. 2). In the GO analysis, 26,567 of the 39,992 nr annotated transcripts had GO terms, which were classified into 60 categories, including cellular process (18,423), binding (17,719), metabolic process (14,226), biological regulation (10,792), regulation of biological process (10,206), catalytic activity (10,202), response to stimulus (8184), and immune system process (1748) (Fig. 3).

### Unigene metabolic pathway analysis

A metabolic pathway analysis of the unigenes was performed using the KEGG annotation system. A total of 28,386 unigenes were mapped to 259 KEGG pathways (Table S3). The metabolic pathway group, which was comprised of 2,946 unigenes (10.38%), represented significantly more unigenes than other pathways, such as those associated with cancer (4.89%), actin cytoskeletal regulation (4.02%), focal adhesion (3.97%), HTLV-I infection (3.46%), endocytosis (3.24%), MAPK signaling (3.15%), influenza A (3.1%), and Epstein–Barr virus infection (3.07%) (Table S3). Among the metabolic pathway group, 40.83% of the unigenes were linked to 35 pathways that were involved in the immunity, autoimmunity diseases, and disease resistance (Table 1), including the cytokine-cytokine receptor interaction pathway that included 433 unigenes. Moreover, 2,335 unigenes (8.22%) participated in six pathways that are associated with bacterial infectious diseases, including pathogenic *Escherichia coli, Salmonella*, and *Vibrio cholerae* infections, shigellosis, pertussis, and the bacterial invasion of epithelial cells (Table S3).

### Identification and annotation of DEGs

**Figure 4:**
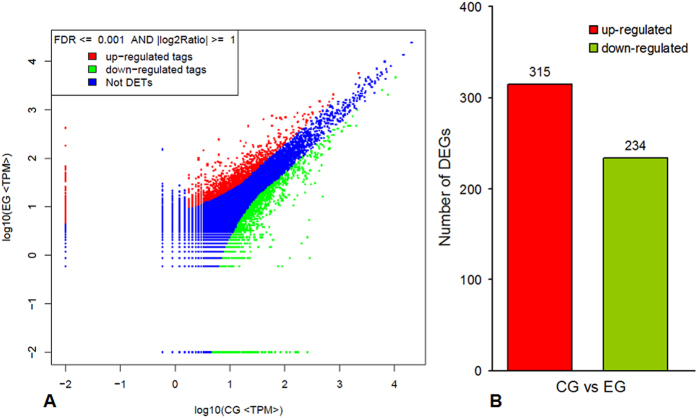
Summary of differentially expressed genes in pairwise comparisons between the mock-challenged control group (CG) and the *A. hydrophila*- challenged experimental group (EG). (**A**) The differential expression analyses of tags by DGE, in which ‘Not DETs’ indicates ‘not detected expression tags’. Limitations are based on FDR ≤ 0.001 and the absolute value of Log2 (EG/CG) being greater than 1. (**B**) The number of differentially expressed genes (CG *vs* EG).

To determine the gene expression profile of the grass carp intestine after being challenged with *A. hydrophila* and to characterize the molecular pathogenesis of the intestinal inflammation caused by *A. hydrophila* infection, two DGE libraries were constructed from *A. hydrophila*-infected and mock-infected grass carp, and 3,578,555 and 3,615,650 raw reads, respectively, were obtained. After removing the low-quality reads, 3,362,986 and 3,404,084 clean reads, respectively, were obtained (Fig. S2). Of these clean reads, 2,328,453 (69.24%) and 2,367,148 (69.54%), respectively, were mapped to gene tags in the mock-challenged control group (CG) and *A. hydrophila*-challenged experimental group (EG) samples, respectively (Table S4). Gene expression levels were normalized to RPKM, and the DEGs were determined based on a Bayesian algorithm. A total of 18,170 genes were shared by the CG and EG samples, of which 549 were differentially expressed in the intestines of these samples. Among these DEGs, 315 were up-regulated and 234 were down-regulated by the *A. hydrophila* challenge (Fig. 4).

### GO analysis of DEGs

**Figure 5:**
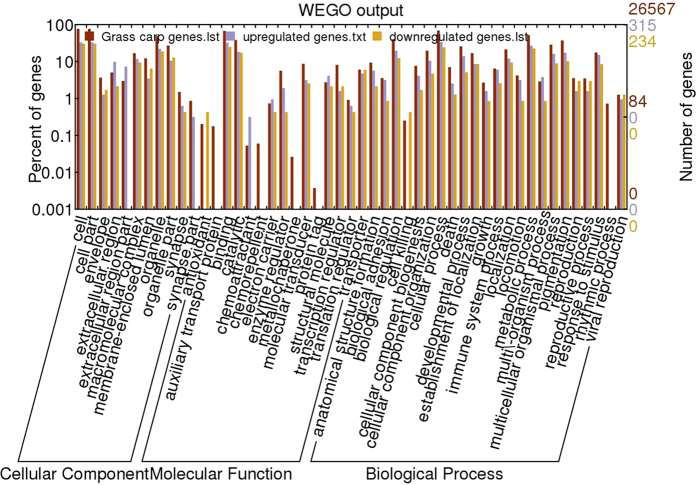
The GO annotations and functional classifications of differentially expressed genes (DEGs) associated with intestinal inflammation in grass carp. GO terms for cellular component, molecular function and biological process are indicated.

The possible DEG functions were determined using the GO classification system. Among the 549 DEGs, 214 (39.0%), 210 (38.3%) and 200 (36.4%) genes were assigned to the biological process, molecular function and cellular component categories, respectively (Fig. 5). The GO enrichment analysis (corrected *p* < 0.05) indicated that 12, 4 and 8 GO terms in biological process, molecular function and cellular component, respectively, were enriched in the DEGs (Table S5). Of these DEGs, 13 (6.1%), 11 (5.1%) and 7 (3.3%) were enriched in immune response, response to bacterium, and the ATP biosynthetic process, respectively, within the biological process ontology, while 18 (8.6%) and 6 (2.9%) were enriched in structural molecule activity and cytokine activity, respectively, within the molecular function ontology. Moreover, 33 (16.5%), 20 (10.0%), 14 (7.0%), and 10 (5.0%) were enriched in extracellular region, extracellular region part, extracellular matrix, and cytosolic part within the cellular component ontology (Table S5).

### KEGG pathways of DEGs

As described above, we identified 549 DEGs. Among these DEGs, 260 were mapped to 165 KEGG pathways. To distinguish the most affected pathways after *A. hydrophila* infection, a KEGG enrichment analysis (corrected *p* < 0.05) was performed. The most significantly enriched KEGG pathways included transport and catabolism (e.g., phagosome and lysosome) in cellular processes, immune diseases (rheumatoid arthritis), and immune system (antigen processing and presentation) (Table S6). Other pathways included energy metabolism (oxidative phosphorylation), endocrine and metabolic diseases (Type I diabetes mellitus), and translation (ribosome) in genetic information processing. The details for these pathways are presented in Figs S3−S9.

### The inflammation-related DEGs in the grass carp intestine

To gain a global view of the DEGs that are associated with the intestinal inflammatory response to bacterial infection, we isolated the inflammation-related KEGG pathways and determined the number of DEGs in each given pathway. The results are summarized in Table 2. The KEGG inflammation-associated pathways were affected by the *A. hydrophila* infection. The most markedly affected pathways, based on the total number of DEGs they contained, included those of the immune system, bacterial infectious diseases, such as pathogenic *E. coli* infections, and signaling molecules and their roles, such as antigen processing and presentation, and cytokine-cytokine receptor interactions. In addition, an *A. hydrophila* infection also affected pathways related to cell communication, cell motility, signal transduction, and immune diseases. Furthermore, notably, the number of genes it up-regulated was larger than, or at least equal to, the number up-regulated in all of the pathways, except chemokine signaling.

### qPCR validation of DEGs

**Figure 6:**
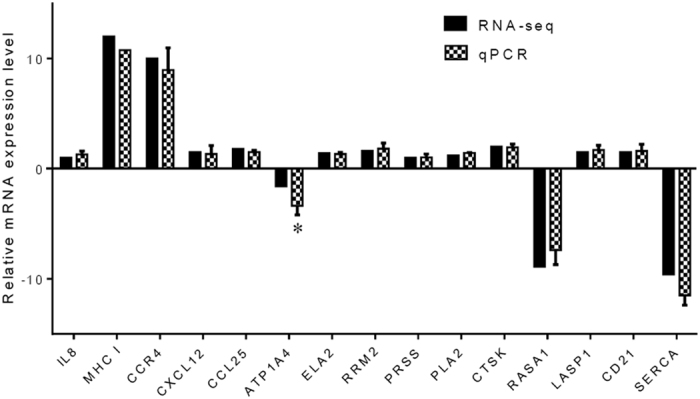
Validation of RNA-seq data by qPCR. To validate the RNA-seq data, the relative mRNA levels of 15 randomly selected DEGs in the intestine of *A. hydrophila*-infected grass carp were examined by qPCR. The mRNA levels by qPCR are presented as the fold change compared with the mock-treated control after normalization against β-actin. The relative expression levels from the RNA-seq analysis were calculated as RPKM values. The asterisk (*) denotes the presence of a statistically significant difference (p < 0.05) in mRNA levels between the RNA-seq and qPCR analysis.

To verify the reliability of the transcriptome data and the DGE results obtained by RNA-seq, 15 randomly selected DEGs, including 12 up-regulated genes (IL-8, major histocompatibility complex class I (MHC I), chemokine (C-C motif) receptor 4 (CCR4), chemokine (C-X-C motif) ligand 12 (CXCL12), chemokine (C-C motif) ligand 25β (CCL25), pancreatic elastase II (ELA2), ribonucleoside-diphosphate reductase subunit M2 (RRM2), trypsinogen (PRSS), phospholipase A2 (PLA2), cathepsin K (CTSK), LIM and SH3 protein 1 (LASP1), and cluster of differentiation 21 (CD21)) and 3 down-regulated genes (Na^+^/K^+^ transporting ATPase subunit α-4 (ATP1A4), RasGTPase-activating protein 1 (RASA1), and Sarco (endo) plasmic reticulum calcium ATPase (SERCA)), were selected for qPCR analysis. As shown in Fig. 6, the fold-change values obtained in qPCR were consistent with the values obtained by RNA-seq for all of the selected genes, except the ATP1A4 gene, which showed significantly different mRNA expression level estimated using these two methods.

### Timing of the intestinal inflammation-related genes’ mRNA expression levels after *A. hydrophila* infection

**Figure 7:**
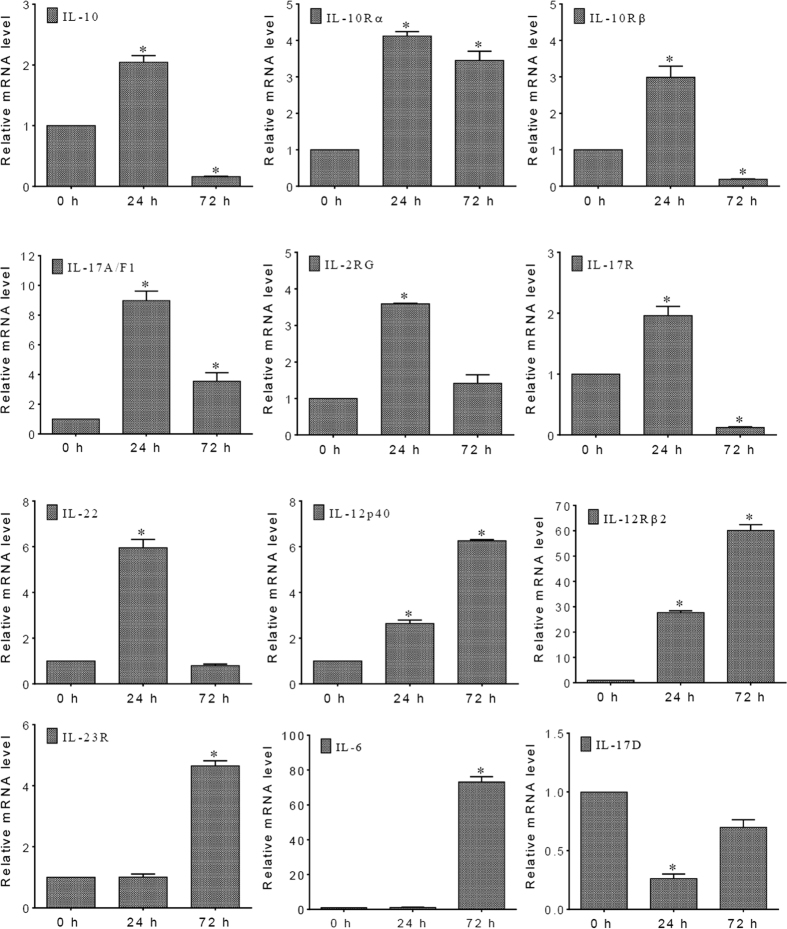
Time course of 12 inflammatory genes’ mRNA expression levels in grass carp intestine after *A. hydrophila* stimulation. The mRNA levels measured by qPCR are presented as the relative fold change normalized against the housekeeping β-actin gene. The horizontal axis in each graph represents the time after *A. hydrophila* stimulation. The asterisk (*) denotes the presence of a statistically significant difference (p < 0.05) at the mRNA level when compared with the control (0 h).

To further understand the regulatory mechanisms of inflammatory cytokines in intestinal inflammation, a time course of 12 intestinal inflammation-related genes’ mRNA expression levels was examined by qPCR analysis. The results are shown in Fig. 7. The gene expression levels could be roughly clustered into four patterns over time. The most common pattern was displayed by seven genes, including IL-10 and its receptors (IL-10Rα and IL-10Rβ), IL-17A/F1 and its receptor (IL-17R), IL-22, and IL-2RG. The mRNA levels of these genes were significantly up-regulated at 24 h following *A. hydrophila* infection, and then decreased at 72 h. The second pattern was observed in two genes, IL-12p40 and IL-12Rβ2, which were progressively up-regulated upon bacterial infection. Furthermore, IL-23R and IL-6 genes were expressed in the third pattern, in which their significant up-regulation lagged considerably. Finally, in contrast to the other genes, IL-17D was significantly down-regulated within 24 h of anal intubation with *A. hydrophila*, but its mRNA level returned to a nearly normal level by 72 h.

## Discussion

Inflammatory diseases, especially bacterial enteritis, are among the most prevalent fish diseases affecting sustainable aquaculture. Bacterial enteritis has long been recognized as a highly prevalent intestinal disease that frequently occurs in both freshwater and marine aquaculture^5^,^38^. However, the pathogenic mechanisms in bacterial-induced fish enteritis and the roles of inflammatory genes in modulating the intestinal inflammation are largely unknown, although the immune responses of grass carp and other farmed fish to bacterial infection have been examined by transcriptome analysis^37^,^38^. The fish intestine is an important mucosal organ and is a major route of bacterial infection. Thus, we are more interested in the genes involved in the intestinal inflammatory response. In our previous study, we constructed a model of intestinal inflammation in grass carp based on infection with pathogenic *A. hydrophila* and found that the inflammatory responses induced by the *A. hydrophila* infection were closely associated with the expression levels of the pro-inflammatory cytokines IL-1β and IL-8, and TNF-α^**7**^. In an effort to further understand the mechanism of *A. hydrophila*-induced intestinal inflammation, in this study, we generated transcriptomic data based on the grass carp model mentioned above. In contrast to other studies that focused on the transcriptomes of the grass carp head kidney, hepatopancreas, and spleen^21^,^22^,^36^,^39^,^40^, the transcriptomic profiles of the intestines from *A. hydrophila*-infected and mock-infected grass carp were compared in this study. Our work also differs from that reported in *Vibrio harveyi*-infected carnivorous Asian seabass^41^.

In this study, 549 DEGS were detected in grass carp intestine after *A. hydrophila* infection. This was a similar amount as identified in the same tissue 48 h after a reovirus infection^42^ and less than the amount found in both spleen and kidney tissues in a study aimed to identify miRNA targets using *A. hydrophila*-susceptible and/or resistant grass carp^36^. It is difficult to determine a precise number of inflammation-related genes among the 549 DEGs, since certain genes (such as IL-8) may participate in multiple KEGG pathways. However, it is evident that the number of up-regulated inflammation-related genes is significantly greater than the number of down-regulated genes. In fact, similar observations have been noted during the inflammatory process in human gastric mucosa following *Helicobacter pylori* infection^43^ and in ovine bone marrow-derived dendritic cells upon stimulation with *Staphylococcus aureus*^44^. With regard to the up-regulation or down-regulation of inflammation-related genes in the KEGG pathways, it is worth noting that a relatively large number of differentially expressed inflammation-related genes are involved in the immune system (see Table 2), which suggests that bacterial infection triggers a great inflammatory immune response in host intestinal cells.

In the present study, 15 DEGs were selected for qPCR to verify the RNA-seq data. Overall, the qPCR assay demonstrated the high level of consistent results between the two methods, and supports the reliability of the RNA-seq data analysis. The 12 genes selected for the time-course analysis are all inflammatory cytokines in the inflammatory bowel disease KEGG pathway. Ten of these 12 genes were identified in the reference transcriptome data generated by RNA-seq in this study, while the other two, IL-17D and IL-17A/F1, were not. However, recent reports confirmed that these two genes are actually expressed in the intestine and other tissues of grass carp^45^,^46^. Although the potential reasons for this are not fully clear, possible explanations might include, but are not limited to: 1) very low mRNA expression levels of these genes in the intestine^46^; and 2) no transcription of these particular genes due to temporal expression patterns. In addition, we failed to detect the BAFF gene, which is involved in inflammatory responses^47^, probably due to its very low expression level in grass carp intestine^48^.

In this study, the mRNA expression levels for all 12 genes were examined over time. These genes could be categorized into four distinct groups based on their different mRNA expression patterns over time during the *A. hydrophila*-induced intestinal inflammation, indicating the complex nature and critical role of the cytokine-mediated signaling network in regulating inflammatory response. Interestingly, among the 12 genes, IL-17D was expressed in a pattern distinctly different from others. A previous study indicated that an intraperitoneal injection with *A. hydrophila* resulted in dramatic increase of the IL-17D mRNA level in grass carp head kidney^45^. However, we found a significant decrease in the IL-17D expression level in intestines when the *A. hydrophila* cells were delivered via anal intubation. This discrepancy might be resolved if IL-17D plays similar, but not identical, roles in intestinal mucosal and systemic immune responses.

Interestingly, the MMP-9 gene, encoding matrix metalloproteinase-9, which is associated with inflammatory processes and plays an important role in *A. hydrophila*-related diseases^49^, was also identified in this study as a DEG after *A. hydrophila* stimulation, and was mapped to three KEGG pathways, cancer, bladder cancer, and leukocyte transendothelial migration. Additionally, a differentially expressed transcript that shares a high sequence homology with zebrafish chemokine (C-X-C motif) ligand 8b, duplicate 3 (cxcl8b.3) was mapped to as many as 14 different pathways, including rheumatoid arthritis, cytokine-cytokine receptor interaction, and multiple signaling pathways (chemokines, Toll-like receptor, NOD-like receptor, and RIG-I-like receptor signaling pathways).

Other inflammation-related genes, such as colony-stimulating factor 1 receptor (CSF1R) and C-X-C chemokine receptor type 5 (CXCR5) (also known as Burkitt lymphoma receptor 1, BLR1), were also detected in this study, although they were not significantly up-regulated by the *A. hydrophila* infection. The CSF1R was mainly expressed in the grass carp spleen, head kidney, and head kidney-derived monocytes/macrophages, and its expression was altered after the *A. hydrophila* infection^50^. The CXCR5 gene plays a critical role in solitary intestinal lymphoid tissue formation^51^. In grass carp, CXCR5 was strongly expressed in intestine and other tissues^52^. We identified CXCR5, but did not find *A. hydrophila*-induced up-regulation. Thus, we propose that, in this study, *A. hydrophila* induced an acute phase inflammatory response in the intestines.

As a critical part of the body’s immune response^53^, inflammation is highly associated with human immune diseases (e.g., rheumatoid arthritis and inflammatory bowel disease), while in fish, inflammation is commonly observed in infectious diseases (bacterial, viral, and even fungal), particularly enteritis^54^. Over the last few years, a considerable amount of fish transcriptome sequence data has accumulated, and a few inflammatory genes and their biological roles have been separately described. Despite these efforts, much work is still necessary to elucidate the exact mechanism underlying the fish inflammatory response to invading pathogens. In this study, we provide novel intestinal transcriptome sequence data with a special focus on the discovery of inflammation-related genes and their signaling networks. These data will allow us to better understand not only the mechanism of intestinal inflammation triggered by bacterial pathogens, but also the pathogenesis of enteritis and other fish inflammatory diseases, like aerocystitis^55^, mycobacteriosis^56^ and glomerulonephritis^57^. Additionally, and more practically, the data might aid in the development of therapeutic targets for fish diseases associated with inflammation^58^.

In conclusion, using the Illumina HiSeq™ 2000 sequencing platform, we generated the intestinal transcriptomes of grass carp infected by *A. hydrophila*, identified a few inflammation-related genes, and examined the time-course mRNA expression patterns of inflammatory cytokines. Our results provide a deep insight into the intestinal inflammatory response of grass carp to *A. hydrophila* infection that will be helpful in identifying reliable therapeutic targets for fish inflammatory diseases.

## Materials and Methods

### Animals and bacterial challenge

Grass carp fingerlings were supplied by the Wujiang Aquaculture Co., Ltd., Jiangsu, China. The fish were cultured at 28 ± 1 °C in re-circulating rearing system consisting of 20 cylindrical tanks (75 cm in diameter). Fifteen fish were kept in each tank containing 60-cm deep filtered and oxygenated water. Each day ~30% of the water was replaced, and the fish were fed a commercial diet (1% body weight). After acclimatization for two weeks, fish showing no abnormal appearance or apparent clinical signs of disease were considered to be healthy. Healthy fish with similar body weights (65.6 ± 7.3 g) were selected for this study. A strain of *A. hydrophila* (CCTCC accession number M2013089) was activated to induce intestinal inflammation in fish^7^. The overnight cultured bacterial cells were harvested by centrifugation, and re-suspended in PSS. After being anesthetized with MS-222, each fish was challenged through anal intubation with 0.3 mL of bacterial suspension (2.1 × 10^7^ cfu/mL). The fish that received the same volume of PSS were the mock-challenged control group. Following anal intubation, different groups of fish were separately kept in tanks with oxygenated water at 28 ± 1 °C.

### Tissue sampling

The thymus, head kidney, spleen, liver, skin, gill, and intestinal tissues were collected from healthy fish for use in *de novo* transcriptome sequencing. For a comparative transcriptome analysis, the intestines were collected from *A. hydrophila*-challenged or mock-challenged fish 24 h after the anal intubation of the bacterial suspension or PSS. Besides, for qPCR analysis, the intestinal samples were also harvested at 0 h, 24 h, and 72 h following challenge. Each sampling was performed from three fish immediately after anesthetization. These samples were stored at –80 °C until RNA isolation. All of the animal experiments were performed in accordance with the Guidelines for Care and Use of Laboratory Animals of Jiangsu Province, China, and the experimental protocols were approved by Soochow University Ethics Committee.

### cDNA library preparation and Illumina sequencing

Total RNA was isolated from the thymus, head kidney, spleen, liver, skin, gill, and intestinal samples using TRIzol reagent following the manufacturer’s protocol (Invitrogen, Carlsbad, CA, USA). The extracted RNA was treated with RNase-free DNase I to eliminate residual genomic DNA contamination. The quality of the RNA was checked using agarose gel electrophoresis and NanoDrop spectrophotometry (NanoDrop ND-1000, Wilmington, DE, USA). After RNA extraction, poly(A)-containing mRNAs were purified using oligo(dT)-attached magnetic beads. Equal amounts of RNA isolated from different samples were mixed. Using the fragmented RNA as the template, first strand cDNA was synthesized using random primers. Second-strand cDNA was synthesized using a buffer containing DNA polymerase I, RNase H, and dNTPs. cDNA fragments were ligated to adapters after the end-repair process. These products were purified and enriched to develop a cDNA library. Finally, the complete library was sequenced by BGI-Shenzhen (Shenzhen, China) using the Illumina HiSeq™ 2000 system (Illumina, San Diego, CA, USA).

### Sequence data processing and analysis

The 200 bp raw paired-end reads generated by the Illumina Genome Analyzer II system were assembled into non-redundant (nr) consensus sequences using TGICL, and CAP3 software. The adaptor sequences were trimmed using the Cross_Match software in the Phrap package (http://www.phrap.org/). Short sequences (<100 bp) were removed using a custom Perl program. The resulting high-quality sequences were assembled into sequence contigs with the TGICL program, which creates an assembly using CAP3. A sequence homology search was performed using local BLASTall programs against sequences in the NCBI nr protein database and the Swissprot database (E-value < 1e^−10^). Genes were tentatively identified according to the best hits against known sequences. Assembled consensus sequences were used to determine the Gene Ontology (GO) (http://www.geneontology.org/) and the Clusters of Orthologous Groups (COGs) terms, and were analyzed further against the Kyoto Encyclopedia of Genes and Genomes Pathway (KEGG) database (http://www.genome.jp/kegg/)^59^.

### Digital gene expression (DGE) tag profiling

Sequence tags were prepared using Illumina’s Digital Gene Expression Tag Profiling Kit (San Diego, CA, USA) according to the manufacturer’s instructions. Then, sequencing was performed using the Illumina Genome Analyzer II system according to the manufacturer’s protocols. Image analyses, base calling, raw 17 bp tag generation and tag counting were performed using the Illumina Analysis Pipeline.

### Aligning the DGE tags to reference transcriptome data

DGE libraries were constructed from the intestines of an EG and a CG. After the DGE tags were assessed for their sequence quality using the Illumina pipeline, clean tags were collected and characterized using custom Perl scripts. All of the clean tags were then aligned to the reference transcriptome data generated by RNA-seq, allowing for only a one nucleotide mismatch. To achieve this, the CATG sites were checked on each mRNA sequence from the transcriptome to construct the reference tag database of 21-bp sequences (4 bp ‘CATG’ +17 bp tag).

### Identification of differentially expressed genes (DEGs)

To examine the expression patterns of unigenes under different treatment conditions, the normalization of gene expression levels was performed using reads per kilobase of exon model per million mapped reads (RPKM) values. The ratio of unigene RPKMs between two samples was set as the fold change when the false discovery rate (FDR) was obtained. Unigenes with fold changes >2 and FDR < 0.001 were defined as DEGs. Possible functions and pathways involving these DEGs were determined using the GO annotation system and KEGG database, respectively. GO terms with a corrected *p* value ≤ 0.05 were designated as significantly enriched for DEGs. Web Gene Ontology Annotation Plot (WEGO) software was used for visualizing, comparing, and plotting GO annotation results. Pathways with a corrected *p* value ≤ 0.05 were designated as significantly enriched pathways for DEGs.

### Quantitative real-time PCR (qPCR) analysis

To validate the RNA-seq data, 15 genes (IL-8, MHC I, CCR4, CXCL12, CCL25, ATP1A4, ELA2, RRM2, PRSS, PLA2, CTSK, RASA1, LASP1, CD21, and SERCA) were randomly chosen from all of the DEGs. The mRNA expression levels of these selected genes were then determined using qPCR. Briefly, total RNA was isolated from the intestines of mock-challenged control fish and those with intestinal inflammation induced by *A. hydrophila* infections, using TRIzol, and reverse-transcribed into cDNA. Following reverse transcription, a qPCR analysis was performed as described previously^4^. The relative expression levels, in fold changes relative to the internal control β-actin, were calculated using the 2^−ΔΔCT^ method. The same procedure was also followed for measuring the expression profiles of 12 selected genes, which were involved in inflammatory bowel disease (KEGG pathway: hsa05321). The oligonucleotide primer pairs used in all of the qPCR reactions are listed in Table S7.

### Statistics

All of the data are presented as mean ± standard deviation (SD). Statistical differences were evaluated using Student’s *t*-test for unpaired samples. The level of a statistically significant difference was set at *p* < 0.05.

## Additional Information

**How to cite this article:** Song, X. *et al*. A transcriptome analysis focusing on inflammation-related genes of grass carp intestines following infection with *Aeromonas hydrophila. Sci. Rep.* **7**, 40777; doi: 10.1038/srep40777 (2017).

**Publisher's note:** Springer Nature remains neutral with regard to jurisdictional claims in published maps and institutional affiliations.

## Supplementary Material

Supplementary Information

Supplementary Dataset 1

Supplementary Dataset 2

## Figures and Tables

**Table 1:** The number of genes categorized into pathways that are directly associated with immune and autoimmune diseases.

Pathway	Number of genes with pathway annotation (28386)	Pathway ID
Endocytosis	921 (3.24%)	ko04144
Chemokine signaling pathway	661 (2.33%)	ko04062
Leukocyte transendothelial migration	595 (2.1%)	ko04670
FcγR-mediated phagocytosis	525 (1.85%)	ko04666
Cell adhesion molecules (CAMs)	504 (1.78%)	ko04514
ECM-receptor interaction	480 (1.69%)	ko04512
Natural killer cell mediated cytotoxicity	440 (1.55%)	ko04650
Cytokine-cytokine receptor interaction	433 (1.53%)	ko04060
T cell receptor signaling pathway	418 (1.47%)	ko04660
NF-κB signaling pathway	416 (1.47%)	ko04064
Systemic lupus erythematosus	263 (0.93%)	ko05322
Antigen processing and presentation	258 (0.91%)	ko04612
Rheumatoid arthritis	212 (0.75%)	ko05323
Intestinal immune network for IgA production	128 (0.45%)	ko04672

**Table 2:** The numbers of differentially expressed intestinal inflammation-related genes in the KEGG pathway.

Category	Pathway	Number of DEGs in pathway	Number of up-regulated DEGs	Number of down-regulated DEGs
Signal transduction	MAPK signaling pathway	2	1	1
	TGF-β signaling pathway	3	2	1
	VEGF signaling pathway	1	1	0
	Jak-STAT signaling pathway	1	1	0
	Calcium signaling pathway	3	0	3
Signaling molecules and interaction	Cytokine-cytokine receptor interaction	10	9	1
	ECM-receptor interaction	6	5	1
	Cell adhesion molecules (CAMs)	9	5	4
Transport and catabolism	Endocytosis	6	4	2
Cell motility	Regulation of actin cytoskeleton	11	8	3
Cell communication	Focal adhesion	11	9	2
	Adherens junction	1	1	0
	Tight junction	5	3	2
Immune system	Toll-like receptor signaling pathway	3	2	1
	NOD-like receptor signaling pathway	2	1	1
	RIG-I-like receptor signaling pathway	1	1	0
	Natural killer cell mediated cytotoxicity	4	4	0
	Antigen processing and presentation	12	10	2
	T cell receptor signaling pathway	1	1	0
	B cell receptor signaling pathway	2	1	1
	FcεRI signaling pathway	1	1	0
	FcγR-mediated phagocytosis	2	2	0
	Leukocyte transendothelial migration	6	4	2
	Intestinal immune network for IgA production	4	2	2
	Chemokine signaling pathway	9	4	5
Immune diseases	Rheumatoid arthritis	11	8	3
Bacterial infectious diseases	Epithelial cell signaling in *Helicobacter pylori* infection	5	4	1
	Pathogenic *Escherichia coli* infection	6	4	2
	Shigellosis	6	4	2
	*Staphylococcus aureus* infection	5	3	2
	*Vibrio cholerae* infection	6	5	1
	Bacterial invasion of epithelial cells	4	3	1
