# Supplementary material for: A transcriptome analysis focusing on inflammation-related genes of grass carp intestines following infection with Aeromonas hydrophila
Source: Sci Rep. 2017 Jan 17;7:40777. doi: 10.1038/srep40777 (PMC5240114; doi:10.1038/srep40777)
Supplement: Supplementary Information [file srep40777-s1.pdf]

A transcriptome analysis focusing on inflammation-related genes of grass carp intestines following infection with *Aeromonas hydrophila*

Xuehong Song[1,#], Xiaolong Hu[1,2,#], Bingyao Sun[1], Yunxuan Bo[1], Kang Wu[1], Lanying Xiao[1], Chengliang Gong[1,2,*]

[1]School of Biology and Basic Medical Sciences, Soochow University, Suzhou 215123, China

[2]National Engineering Laboratory for Modern Silk, Soochow University, Suzhou 215123, China

[*] Corresponding author: gongcl@suda.edu.cn

List of Supplementary Information (Supplementary Tables and Figures)

Table S1. Summary of sequencing data generated for grass carp transcriptome.

Table S2. Summary of *de novo* grass carp transcriptome assembly results.

Table S3. KEGG pathway analysis of the unigenes identified in the grass carp transcriptome.

Table S4. Summary of the RNA-seq alignment statistics in the two libraries.

Table S5. Gene ontology (GO) classification of the significantly differentially expressed genes.

Table S6.The significantly enriched KEGG pathways affected by *A. hydrophila* infection.

Table S7. The sequences of oligonucleotide primer pairs used in quantitative real-time PCR (qPCR).

Figure S1. The size distribution of assembled contigs (A) and unigenes (B) from the grass carp transcriptome.

Figure S2. Distribution of total clean tags from DGE libraries.

Figure S3. The significantly DEG-enriched KEGG phagosome pathway.

Figure S4. The significantly DEG-enriched KEGG lysosome pathway.

Figure S5. The significantly DEG-enriched KEGG rheumatoid arthritis pathway.

Figure S6. The significantly DEG-enriched KEGG antigen processing and presentation pathway.

Figure S7. The significantly DEG-enriched KEGG oxidative phosphorylation pathway.

Figure S8. The significantly DEG-enriched KEGG type I diabetes mellitus pathway.

Figure S9. The significantly DEG-enriched KEGG ribosome pathway.

Table S1. Summary of sequencing data generated for grass carp transcriptome.

Total clean reads	Total clean nucleotides (nt)	Q20 percentage	N percentage	GC percentage
45,867,282	4,128,055,380	95.32%	0.00%	46.61%

Table S2. Summary of *de novo* grass carp transcriptome assembly results.

Sequence type	Total number	Total length (nt)	Mean length (nt)	N50	Total consensus sequences	Distinct clusters	Distinct singletons
Contig	120,964	44,621,250	369	614	-	-	-
Unigene	67,413	44,810,194	665	983	67,413	11,623	55,790

Table S3. KEGG pathway analysis of the unigenes identified in the grass carp transcriptome.

#	Pathway	number of unigenes with pathway annotation (28386 in total)	Pathway ID
1	Metabolic pathways	2946 (10.38%)	ko01100
2	Pathways in cancer	1388 (4.89%)	ko05200
3	Regulation of actin cytoskeleton	1140 (4.02%)	ko04810
4	Focal adhesion	1126 (3.97%)	ko04510
5	HTLV-I infection	983 (3.46%)	ko05166
6	Endocytosis	921 (3.24%)	ko04144
7	MAPK signaling pathway	894 (3.15%)	ko04010
8	Influenza A	879 (3.1%)	ko05164
9	Epstein-Barr virus infection	872 (3.07%)	ko05169
10	Herpes simplex infection	844 (2.97%)	ko05168
11	Tight junction	810 (2.85%)	ko04530
12	Transcriptional misregulation in cancer	782 (2.75%)	ko05202
13	Tuberculosis	744 (2.62%)	ko05152
14	Vascular smooth muscle contraction	679 (2.39%)	ko04270
15	Chemokine signaling pathway	661 (2.33%)	ko04062
16	RNA transport	649 (2.29%)	ko03013
17	Amoebiasis	642 (2.26%)	ko05146
18	Phagosome	641 (2.26%)	ko04145
19	Axon guidance	611 (2.15%)	ko04360
20	Purine metabolism	606 (2.13%)	ko00230
21	Adherens junction	599 (2.11%)	ko04520
22	Leukocyte transendothelial migration	595 (2.1%)	ko04670
23	Wnt signaling pathway	593 (2.09%)	ko04310
24	Insulin signaling pathway	591 (2.08%)	ko04910
25	Spliceosome	542 (1.91%)	ko03040
26	Huntington's disease	542 (1.91%)	ko05016

27	Calcium signaling pathway	530 (1.87%)	ko04020
28	Fc gamma R-mediated phagocytosis	525 (1.85%)	ko04666
29	Toxoplasmosis	516 (1.82%)	ko05145
30	Protein processing in endoplasmic reticulum	516 (1.82%)	ko04141
31	Ubiquitin mediated proteolysis	513 (1.81%)	ko04120
32	Dilated cardiomyopathy	512 (1.8%)	ko05414
33	Cell adhesion molecules (CAMs)	504 (1.78%)	ko04514
34	Viral myocarditis	493 (1.74%)	ko05416
35	Salmonella infection	485 (1.71%)	ko05132
36	ECM-receptor interaction	480 (1.69%)	ko04512
37	Neurotrophin signaling pathway	477 (1.68%)	ko04722
38	Hypertrophic cardiomyopathy (HCM)	468 (1.65%)	ko05410
39	Alzheimer's disease	460 (1.62%)	ko05010
40	Measles	456 (1.61%)	ko05162
41	Natural killer cell mediated cytotoxicity	440 (1.55%)	ko04650
42	Cell cycle	438 (1.54%)	ko04110
43	Cytokine-cytokine receptor interaction	433 (1.53%)	ko04060
44	T cell receptor signaling pathway	418 (1.47%)	ko04660
45	NF-kappa B signaling pathway	416 (1.47%)	ko04064
46	Osteoclast differentiation	413 (1.45%)	ko04380
47	Pathogenic Escherichia coli infection	412 (1.45%)	ko05130
48	Dopaminergic synapse	412 (1.45%)	ko04728
49	Phosphatidylinositol signaling system	408 (1.44%)	ko04070
50	Hepatitis C	401 (1.41%)	ko05160
51	Lysine degradation	398 (1.4%)	ko00310
52	Small cell lung cancer	398 (1.4%)	ko05222
53	Prostate cancer	395 (1.39%)	ko05215
54	Jak-STAT signaling pathway	394 (1.39%)	ko04630
55	Pertussis	393 (1.38%)	ko05133
56	Bacterial invasion of epithelial cells	392 (1.38%)	ko05100

57	Lysosome	387 (1.36%)	ko04142
58	Pancreatic secretion	383 (1.35%)	ko04972
59	Melanogenesis	378 (1.33%)	ko04916
60	Pyrimidine metabolism	377 (1.33%)	ko00240
61	mRNA surveillance pathway	371 (1.31%)	ko03015
62	B cell receptor signaling pathway	368 (1.3%)	ko04662
63	GnRH signaling pathway	362 (1.28%)	ko04912
64	Oocyte meiosis	359 (1.26%)	ko04114
65	Salivary secretion	354 (1.25%)	ko04970
66	TGF-beta signaling pathway	354 (1.25%)	ko04350
67	Renal cell carcinoma	352 (1.24%)	ko05211
68	Chagas disease (American trypanosomiasis)	347 (1.22%)	ko05142
69	ErbB signaling pathway	346 (1.22%)	ko04012
70	Cardiac muscle contraction	344 (1.21%)	ko04260
71	Protein digestion and absorption	343 (1.21%)	ko04974
72	Cholinergic synapse	338 (1.19%)	ko04725
73	Gastric acid secretion	336 (1.18%)	ko04971
74	Vibrio cholerae infection	335 (1.18%)	ko05110
75	Gap junction	333 (1.17%)	ko04540
76	Neuroactive ligand-receptor interaction	322 (1.13%)	ko04080
77	NOD-like receptor signaling pathway	321 (1.13%)	ko04621
78	Long-term potentiation	320 (1.13%)	ko04720
79	Fc epsilon RI signaling pathway	319 (1.12%)	ko04664
80	Chronic myeloid leukemia	319 (1.12%)	ko05220
81	Shigellosis	318 (1.12%)	ko05131
82	Progesterone-mediated oocyte maturation	316 (1.11%)	ko04914
83	Alcoholism	315 (1.11%)	ko05034
84	Complement and coagulation cascades	313 (1.1%)	ko04610
85	Serotonergic synapse	311 (1.1%)	ko04726
86	Leishmaniasis	308 (1.09%)	ko05140

87	Amyotrophic lateral sclerosis (ALS)	300 (1.06%)	ko05014
88	Notch signaling pathway	299 (1.05%)	ko04330
89	VEGF signaling pathway	296 (1.04%)	ko04370
90	Glutamatergic synapse	296 (1.04%)	ko04724
91	Hematopoietic cell lineage	293 (1.03%)	ko04640
92	Arrhythmogenic right ventricular cardiomyopathy (ARVC)	293 (1.03%)	ko05412
93	Apoptosis	292 (1.03%)	ko04210
94	Adipocytokine signaling pathway	288 (1.01%)	ko04920
95	Bile secretion	286 (1.01%)	ko04976
96	Inositol phosphate metabolism	281 (0.99%)	ko00562
97	Ribosome biogenesis in eukaryotes	275 (0.97%)	ko03008
98	Toll-like receptor signaling pathway	273 (0.96%)	ko04620
99	Pancreatic cancer	265 (0.93%)	ko05212
100	Systemic lupus erythematosus	263 (0.93%)	ko05322
101	Glioma	261 (0.92%)	ko05214
102	Colorectal cancer	258 (0.91%)	ko05210
103	Antigen processing and presentation	258 (0.91%)	ko04612
104	p53 signaling pathway	250 (0.88%)	ko04115
105	Glycerophospholipid metabolism	248 (0.87%)	ko00564
106	Ribosome	244 (0.86%)	ko03010
107	Retrograde endocannabinoid signaling	243 (0.86%)	ko04723
108	Peroxisome	242 (0.85%)	ko04146
109	Staphylococcus aureus infection	240 (0.85%)	ko05150
110	Legionellosis	238 (0.84%)	ko05134
111	RNA degradation	236 (0.83%)	ko03018
112	mTOR signaling pathway	235 (0.83%)	ko04150
113	PPAR signaling pathway	232 (0.82%)	ko03320
114	Long-term depression	229 (0.81%)	ko04730
115	Parkinson's disease	224 (0.79%)	ko05012
116	Endometrial cancer	219 (0.77%)	ko05213

117	Epithelial cell signaling in Helicobacter pylori infection	217 (0.76%)	ko05120
118	Acute myeloid leukemia	215 (0.76%)	ko05221
119	Rheumatoid arthritis	212 (0.75%)	ko05323
120	GABAergic synapse	210 (0.74%)	ko04727
121	Amino sugar and nucleotide sugar metabolism	203 (0.72%)	ko00520
122	Morphine addiction	203 (0.72%)	ko05032
123	Non-small cell lung cancer	201 (0.71%)	ko05223
124	Basal cell carcinoma	197 (0.69%)	ko05217
125	RIG-I-like receptor signaling pathway	196 (0.69%)	ko04622
126	Prion diseases	194 (0.68%)	ko05020
127	Melanoma	192 (0.68%)	ko05218
128	Amphetamine addiction	192 (0.68%)	ko05031
129	RNA polymerase	188 (0.66%)	ko03020
130	ABC transporters	185 (0.65%)	ko02010
131	Oxidative phosphorylation	184 (0.65%)	ko00190
132	Mineral absorption	183 (0.64%)	ko04978
133	Autoimmune thyroid disease	183 (0.64%)	ko05320
134	Hedgehog signaling pathway	181 (0.64%)	ko04340
135	Endocrine and other factor-regulated calcium reabsorption	180 (0.63%)	ko04961
136	Allograft rejection	179 (0.63%)	ko05330
137	Glycolysis / Gluconeogenesis	179 (0.63%)	ko00010
138	Starch and sucrose metabolism	176 (0.62%)	ko00500
139	Fructose and mannose metabolism	176 (0.62%)	ko00051
140	Type II diabetes mellitus	174 (0.61%)	ko04930
141	Carbohydrate digestion and absorption	172 (0.61%)	ko04973
142	Aldosterone-regulated sodium reabsorption	169 (0.6%)	ko04960
143	Type I diabetes mellitus	168 (0.59%)	ko04940
144	Arginine and proline metabolism	166 (0.58%)	ko00330
145	Cytosolic DNA-sensing pathway	153 (0.54%)	ko04623
146	Graft-versus-host disease	153 (0.54%)	ko05332

147 Glycerolipid metabolism	152 (0.54%)	ko00561
148 Synaptic vesicle cycle	151 (0.53%)	ko04721
149 Bladder cancer	148 (0.52%)	ko05219
150 Pyruvate metabolism	146 (0.51%)	ko00620
151 Vasopressin-regulated water reabsorption	144 (0.51%)	ko04962
152 Malaria	142 (0.5%)	ko05144
153 Thyroid cancer	138 (0.49%)	ko05216
154 Basal transcription factors	137 (0.48%)	ko03022
155 African trypanosomiasis	135 (0.48%)	ko05143
156 Olfactory transduction	134 (0.47%)	ko04740
157 Arachidonic acid metabolism	133 (0.47%)	ko00590
158 Other types of O-glycan biosynthesis	132 (0.47%)	ko00514
159 Cysteine and methionine metabolism	130 (0.46%)	ko00270
160 Propanoate metabolism	129 (0.45%)	ko00640
161 Cocaine addiction	128 (0.45%)	ko05030
162 Intestinal immune network for IgA production	128 (0.45%)	ko04672
163 Dorso-ventral axis formation	127 (0.45%)	ko04320
164 Sphingolipid metabolism	127 (0.45%)	ko00600
165 Primary immunodeficiency	124 (0.44%)	ko05340
166 Fat digestion and absorption	123 (0.43%)	ko04975
167 N-Glycan biosynthesis	122 (0.43%)	ko00510
168 Fanconi anemia pathway	120 (0.42%)	ko03460
169 Nicotinate and nicotinamide metabolism	120 (0.42%)	ko00760
170 Nucleotide excision repair	117 (0.41%)	ko03420
171 Vitamin digestion and absorption	116 (0.41%)	ko04977
172 Fatty acid metabolism	114 (0.4%)	ko00071
173 Phototransduction - fly	114 (0.4%)	ko04745
174 Circadian rhythm - mammal	114 (0.4%)	ko04710
175 Drug metabolism - other enzymes	113 (0.4%)	ko00983
176 Aminoacyl-tRNA biosynthesis	111 (0.39%)	ko00970

177	Glutathione metabolism	110 (0.39%)	ko00480
178	Valine, leucine and isoleucine degradation	110 (0.39%)	ko00280
179	Pentose and glucuronate interconversions	109 (0.38%)	ko00040
180	Tryptophan metabolism	107 (0.38%)	ko00380
181	Metabolism of xenobiotics by cytochrome P450	106 (0.37%)	ko00980
182	Retinol metabolism	106 (0.37%)	ko00830
183	Steroid hormone biosynthesis	104 (0.37%)	ko00140
184	Proximal tubule bicarbonate reclamation	101 (0.36%)	ko04964
185	Base excision repair	99 (0.35%)	ko03410
186	Glycine, serine and threonine metabolism	97 (0.34%)	ko00260
187	Porphyrin and chlorophyll metabolism	96 (0.34%)	ko00860
188	Drug metabolism - cytochrome P450	95 (0.33%)	ko00982
189	Galactose metabolism	91 (0.32%)	ko00052
190	Ascorbate and aldarate metabolism	91 (0.32%)	ko00053
191	Ether lipid metabolism	90 (0.32%)	ko00565
192	Asthma	88 (0.31%)	ko05310
193	Alanine, aspartate and glutamate metabolism	87 (0.31%)	ko00250
194	Proteasome	87 (0.31%)	ko03050
195	MAPK signaling pathway - fly	84 (0.3%)	ko04013
196	DNA replication	83 (0.29%)	ko03030
197	Citrate cycle (TCA cycle)	78 (0.27%)	ko00020
198	Phototransduction	76 (0.27%)	ko04744
199	Linoleic acid metabolism	72 (0.25%)	ko00591
200	beta-Alanine metabolism	72 (0.25%)	ko00410
201	Collecting duct acid secretion	71 (0.25%)	ko04966
202	Pentose phosphate pathway	71 (0.25%)	ko00030
203	Taste transduction	66 (0.23%)	ko04742
204	Fatty acid elongation	65 (0.23%)	ko00062
205	Glyoxylate and dicarboxylate metabolism	64 (0.23%)	ko00630
206	Mismatch repair	64 (0.23%)	ko03430

207	Circadian rhythm - fly	64 (0.23%)	ko04711
208	Glycosylphosphatidylinositol(GPI)-anchor biosynthesis	62 (0.22%)	ko00563
209	Homologous recombination	60 (0.21%)	ko03440
210	Tyrosine metabolism	60 (0.21%)	ko00350
211	Glycosaminoglycan biosynthesis - heparan sulfate	59 (0.21%)	ko00534
212	Biosynthesis of unsaturated fatty acids	58 (0.2%)	ko01040
213	Regulation of autophagy	57 (0.2%)	ko04140
214	SNARE interactions in vesicular transport	57 (0.2%)	ko04130
215	Butanoate metabolism	56 (0.2%)	ko00650
216	Fatty acid biosynthesis	55 (0.19%)	ko00061
217	Histidine metabolism	55 (0.19%)	ko00340
218	Mucin type O-Glycan biosynthesis	55 (0.19%)	ko00512
219	Other glycan degradation	53 (0.19%)	ko00511
220	Glycosaminoglycan biosynthesis - chondroitin sulfate	53 (0.19%)	ko00532
221	Non-homologous end-joining	53 (0.19%)	ko03450
222	alpha-Linolenic acid metabolism	49 (0.17%)	ko00592
223	Pantothenate and CoA biosynthesis	49 (0.17%)	ko00770
224	Primary bile acid biosynthesis	48 (0.17%)	ko00120
225	Glycosaminoglycan degradation	46 (0.16%)	ko00531
226	Glycosphingolipid biosynthesis - lacto and neolacto series	46 (0.16%)	ko00601
227	One carbon pool by folate	44 (0.16%)	ko00670
228	Selenocompound metabolism	43 (0.15%)	ko00450
229	Terpenoid backbone biosynthesis	39 (0.14%)	ko00900
230	Renin-angiotensin system	37 (0.13%)	ko04614
231	Maturity onset diabetes of the young	37 (0.13%)	ko04950
232	Phenylalanine metabolism	36 (0.13%)	ko00360
233	Steroid biosynthesis	36 (0.13%)	ko00100
234	Protein export	35 (0.12%)	ko03060
235	Glycosphingolipid biosynthesis - globo series	33 (0.12%)	ko00603
236	Glycosaminoglycan biosynthesis - keratan sulfate	33 (0.12%)	ko00533

237	Sulfur relay system	32 (0.11%)	ko04122
238	Glycosphingolipid biosynthesis - ganglio series	30 (0.11%)	ko00604
239	Sulfur metabolism	28 (0.1%)	ko00920
240	Riboflavin metabolism	27 (0.1%)	ko00740
241	Folate biosynthesis	27 (0.1%)	ko00790
242	Cyanoamino acid metabolism	27 (0.1%)	ko00460
243	Ubiquinone and other terpenoid-quinone biosynthesis	25 (0.09%)	ko00130
244	Taurine and hypotaurine metabolism	24 (0.08%)	ko00430
245	Nicotine addiction	19 (0.07%)	ko05033
246	Synthesis and degradation of ketone bodies	14 (0.05%)	ko00072
247	Vitamin B6 metabolism	13 (0.05%)	ko00750
248	Thiamine metabolism	12 (0.04%)	ko00730
249	D-Glutamine and D-glutamate metabolism	11 (0.04%)	ko00471
250	D-Arginine and D-ornithine metabolism	11 (0.04%)	ko00472
251	Phenylalanine, tyrosine and tryptophan biosynthesis	10 (0.04%)	ko00400
252	Butirosin and neomycin biosynthesis	10 (0.04%)	ko00524
253	Valine, leucine and isoleucine biosynthesis	8 (0.03%)	ko00290
254	Caffeine metabolism	8 (0.03%)	ko00232
255	Lipoic acid metabolism	7 (0.02%)	ko00785
256	Biotin metabolism	6 (0.02%)	ko00780
257	Polyketide sugar unit biosynthesis	4 (0.01%)	ko00523
258	Insect hormone biosynthesis	2 (0.01%)	ko00981
259	Lysine biosynthesis	2 (0.01%)	ko00300

Table S4. Summary of the RNA-seq alignment statistics in the two libraries.

Samples	Raw tags	Distinct tags	Clean tags	Distinct clean tags	Map to gene tags	Unique map to gene tags	Unknown tags
CG	3578555	322668	3362986	119479	2623920(78.02%)	2328453(69.24%)	739066(21.98%)
EG	3615650	316393	3404084	116890	2677430(78.65%)	2367148(69.54%)	726654(21.35%)

Table S5. Gene ontology (GO) classification of the significantly differentially expressed genes.

Gene Ontology term	Cluster frequency	Genome frequency of use	Corrected P-value
**Cellular Component**	Cluster frequency (200)	Genome frequency of use (21131)	
extracellular region	33 (16.5%)	1267 (6.0%)	1.92E-05
extracellular region part	20 (10.0%)	721 (3.4%)	0.0031
extracellular matrix	14 (7.0%)	417 (2.0%)	0.00801
hemoglobin complex	3 (1.5%)	9 (0.0%)	0.01163
cytosolic part	10 (5.0%)	230 (1.1%)	0.01231
cytosolic ribosome	6 (3.0%)	82 (0.4%)	0.02222
MHC protein complex	5 (2.5%)	54 (0.3%)	0.02712
proton-transporting two-sector ATPase complex	5 (2.5%)	58 (0.3%)	0.03811
**Molecular Function**	Cluster frequency (210)	Genome frequency of use (22432)	
cytokine activity	6 (2.9%)	56 (0.2%)	0.00292
oxygen transporter activity	3 (1.4%)	12 (0.1%)	0.03544
structural molecule activity	18 (8.6%)	730 (3.3%)	0.03959
monocarboxylic acid transmembrane transporter activity	4 (1.9%)	31 (0.1%)	0.04083
**Biological Process**	Cluster frequency (214)	Genome frequency of use (21350)	
regulation of transcription involved in G1/S phase of mitotic cell cycle	3 (1.4%)	10 (0.0%)	0.010034
response to bacterium	11 (5.1%)	278 (1.3%)	0.010739
energy coupled proton transport, down electrochemical gradient	4 (1.9%)	32 (0.1%)	0.025118
ATP synthesis coupled proton transport	4 (1.9%)	32 (0.1%)	0.025118
prostaglandin transport	2 (0.9%)	3 (0.0%)	0.026434
gas transport	3 (1.4%)	15 (0.1%)	0.036665
oxygen transport	3 (1.4%)	15 (0.1%)	0.036665
viral genome expression	5 (2.3%)	63 (0.3%)	0.037538
viral transcription	5 (2.3%)	63 (0.3%)	0.037538
translational termination	5 (2.3%)	65 (0.3%)	0.043404
immune response	13 (6.1%)	440 (2.1%)	0.046614
ATP biosynthetic process	7 (3.3%)	140 (0.7%)	0.047625

Table S6. The significantly enriched KEGG pathways affected by *A. hydrophila* infection.

Pathway	DEGs with pathway annotation (260)	Genes with pathway annotation (28386)	P-value	Q-value	Pathway ID
Rheumatoid arthritis	11 (4.23%)	212 (0.75%)	4.56E-06	0.000428	ko05323
Antigen processing and presentation	12 (4.62%)	258 (0.91%)	5.18E-06	0.000428	ko04612
Phagosome	16 (6.15%)	641 (2.26%)	0.00030861	0.012821	ko04145
Oxidative phosphorylation	8 (3.08%)	184 (0.65%)	0.00031082	0.012821	ko00190
Type I diabetes mellitus	7 (2.69%)	168 (0.59%)	0.00094356	0.026215	ko04940
Lysosome	11 (4.23%)	387 (1.36%)	0.00095326	0.026215	ko04142
Ribosome	8 (3.08%)	244 (0.86%)	0.00193558	0.045624	ko03010

Note: Only the Q-values for pathways considered as significantly enriched (Q-value < 0.05) were shown in the table.

Table S7. The sequences of oligonucleotide primer pairs used in quantitative real-time PCR (qPCR).

Gene names	Upstream primers	Downstream primers
IL-8	qIL8F: GCTGCTAGACTGTTTGCTCCAC	qIL8R: GAGGCAGGTCTGTCCTTGTTTC
MHC I	qMHCF: TGGAGCAGCGGCAAACTAC	qMHCR: TTGGGTAGAATCCTGTGGCA
CCR4	qCCRF: ATGGTAGGGAAACATCGCACT	qCCRR: GCAGAAATACAAGCGAGCGA
CXCL12	qCXCLF: GCTCAAGTTCCTCCACACACC	qCXCLR: GACGGTGGGCTGTCAGATTC
CCL25	qCCLF: TAACTAAACAGAGGAGGAGACATAAGC	qCCLR: CAGTTTTCATAGGAACCTTGTGC
ATP1A4	qATPF: GTTCTTCTTTGCCATTCGTTTG	qATPR: AGCACTTCATCCACATCATCACT
ELA2	qELAF: GTCAAGGAATCTATGGTCTGTGC	qELAR: GGACACACGGGTAAAGACAGTAG
RRM2	qRRMF: TTGGTGGAACGATTTACTCAGG	qRRMR: ACAAGGCATTGTCTCAATAGCA
PRSS	qPRSSF: ATGGTATTGTGTCCTGGGGTT	qPRSSR: TTAGTTGCTGCTTATGGTGTCG
PLA2	qPLAF: CCCTACAGCAATCCCACCTC	qPLAR: TTGGCTCTCACGCAATCTTC
CTSK	qCTSKF: TTACGGAGTCACACCGAAAGG	qCTSKR: TTACGAGCCATCAGGACATACC
RASA1	qRASAF: CAAACGCTTGTTGAATGTGGT	qRASAR: CAGATTGTGATTCAGCACTTTAGC
LASP1	qLASPF: CGTTTCTTCACCTTATTCCTTCG	qLASPR: CAGTATGGTCTCTTCTCAAAGCCTT
CD21	qCDF: CAATGCCACACTACTGTCCTACC	qCDR: GACCCATAGAGACGGTATCCACT
SERCA	qSERCAF: AGCGACAGCAACTCCAATAGC	qSERCAR: GATGCTGTCCCCGACCTTAG
IL-6	qIL6F: CAACGACATCAAACACGAGACC	qIL6R: TGTCCACCCTTCCTCTTGCT
IL-10	qIL10F: GGATGTTGCTGTGGATTGGA	qIL10R: TGCCTTATCCTACAGTATGTGGTT
IL10Rα	q10RAF: TCCGTGTGAAAGTGGAGATGAC	q10RAR: GGATTTCAGAACAGCAGGCAT

IL10Rβ	q10RBF: ACTGACAGCAGCAGGGAGAAG	q10RBR: AGCAGAACCGAAAGAAAGCC
IL-12p40	qp40F: AGGAGCATCAATCTCACTCTGTTC	qp40R: GCCACTCAAATACATCATCTTCG
IL12Rβ2	q12RBF: CTCTTGAGGGATTTCAGACCATT	q12RBR: CACACATTAGAGGCATCTCCACA
IL-17R	qIL17RF: TCTCTGGCTGGACTCTTGGAT	qIL17RR: GAGGAGGATGTGAGAGGAGAAAC
IL2RG	qIL2RGF: GGTTGGAGAGGATGCTTTCAC	qIL2RGR: GCCTGTCCTGTGCGTGAGTA
IL-22	qIL22F: GTCGTGTTTCGTGGTCTTTGTC	qIL22R: TGTGCTGCTGGTGCTTTCTG
IL-17A/F1	qAF1F : GGACCTACACGTTTACGCATG	qAF1R: ACAGGTAATCCTCACTGCCATC
IL-17D	q17DF: ACCTGTCAATCAGCGTCTCG	q17DR: CTGGATCATGGGAGATTCTGTAC
IL23R	q23RF: GCCGATGATTCCAACTATTTACAC	q23RR: TGACCTGTGTTGAAGACAGACTCT
β-actin	actF: CCTTCTTGGGTATGGAGTCTTG	actR: AGAGTATTTACGCTCAGGTGGG

Notes: IL-8: interleukin-8; MHC I: major histocompatibility complex, class I; CCR4: chemokine (C-C motif) receptor 4; CXCL12: chemokine (C-X-C motif) ligand 12; CCL25: chemokine (C-C motif) ligand 25β; ATP1A4: Na^+/K^+ transporting ATPase subunit α-4; ELA2: pancreatic elastase II; RRM2: ribonucleoside-diphosphate reductase subunit M2; PRSS: trypsinogen; PLA2: phospholipase A2; CTSK: cathepsin K; RASA1: RasGTPase-activating protein 1; LASP1: LIM and SH3 protein 1; CD21: cluster of differentiation 21; SERCA: Sarco (endo) plasmic reticulum calcium ATPase; IL-6: interleukin-6; IL-10: interleukin-10; IL10RA: interleukin-10 receptor α; IL10RB: interleukin-10 receptor β; IL-12p40: interleukin-12 p40; IL12RB2: interleukin-12 receptor β-2; IL17R: interleukin-17 receptor; IL-17A/F1: interleukin-17A/F1; IL2RG: interleukin-2 receptor γ; IL-22: interleukin-22; IL-17D: interleukin-17D; IL-23R: interleukin-23 receptor.

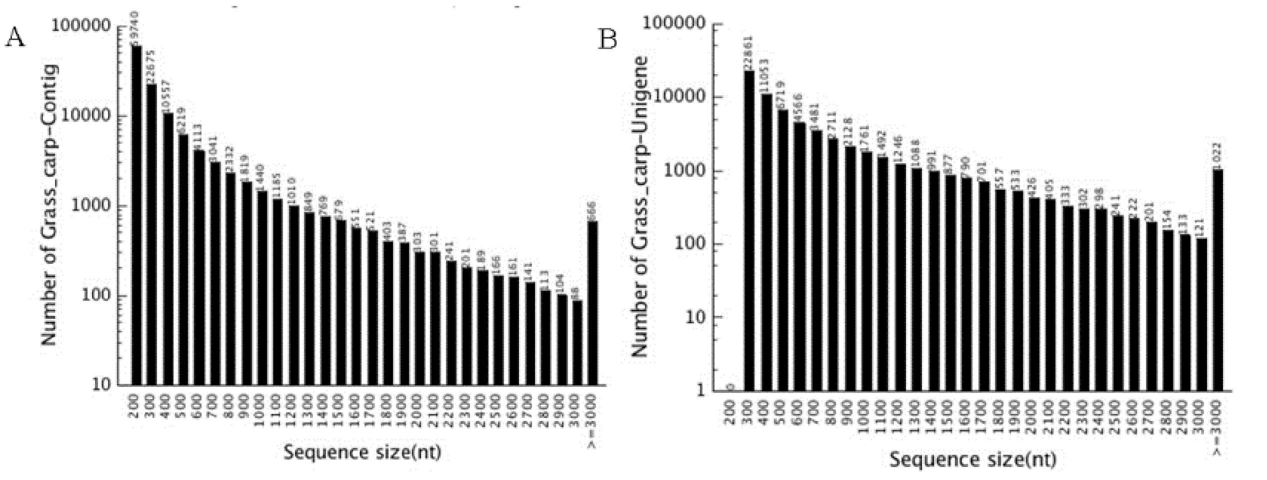

Figure S1. The size distribution of assembled contigs (A) and unigenes (B) from the grass carp transcriptome.

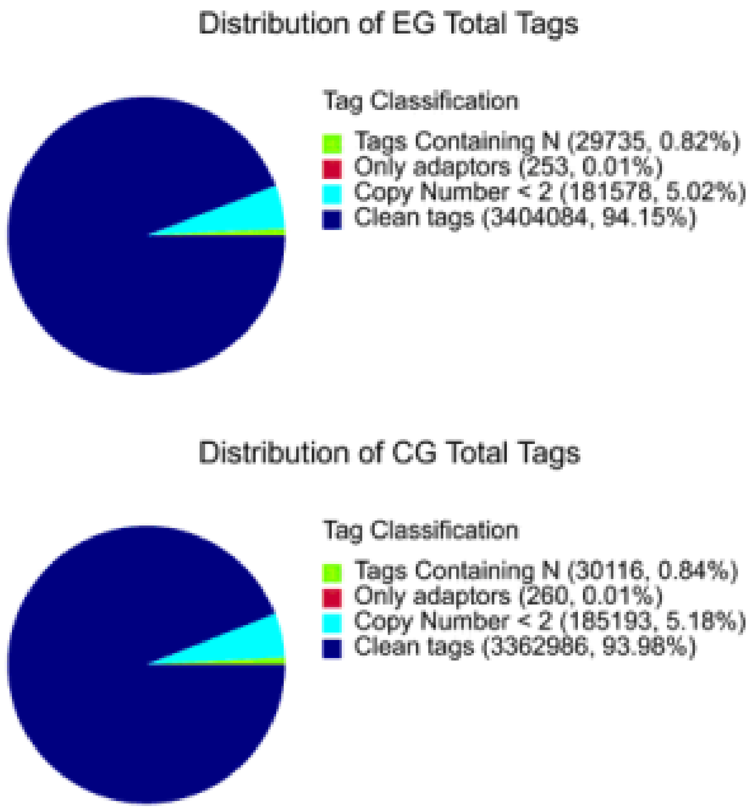

Figure S2. Distribution of total clean tags from DGE libraries.

The numbers (and the percentage in parentheses) indicate each tag class (including tags containing N, empty tags with the adaptor only, tags with a copy number < 2, and clean tags) number out of the total number of tags. Data from the experimental group (top) and control group (bottom) are shown.

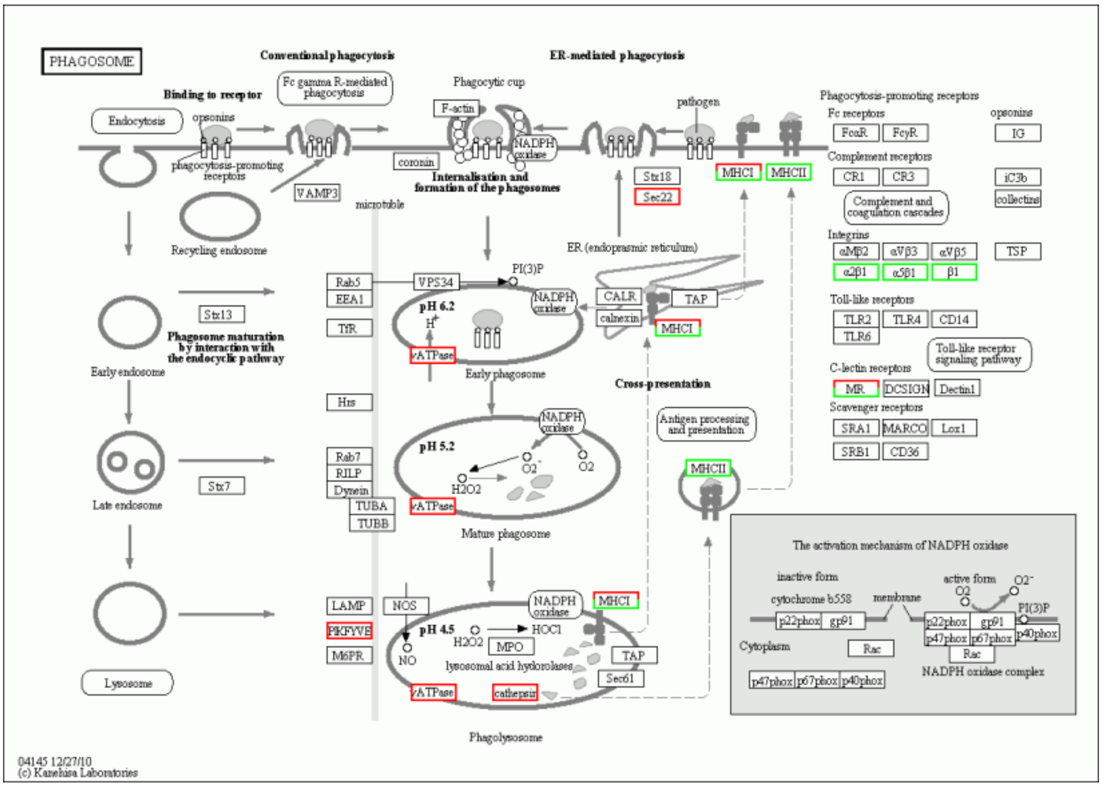

Figure S3. The significantly DEG-enriched KEGG phagosome pathway.

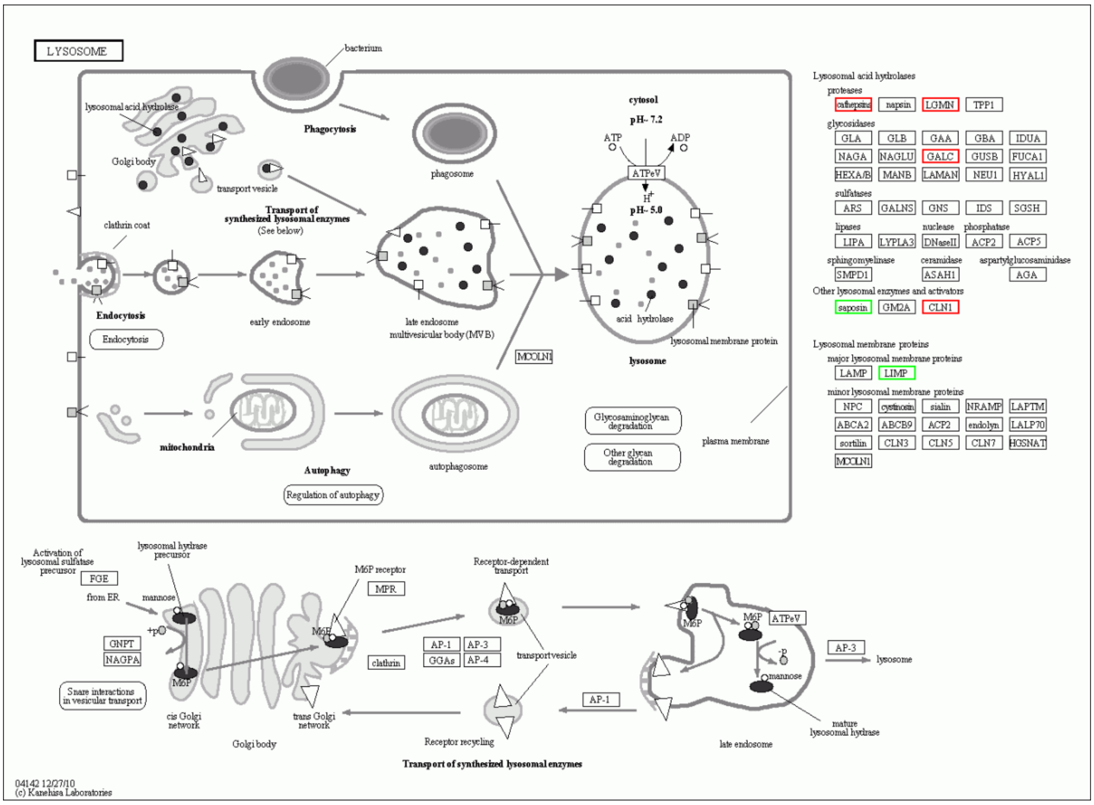

Figure S4. The significantly DEG-enriched KEGG lysosome pathway.

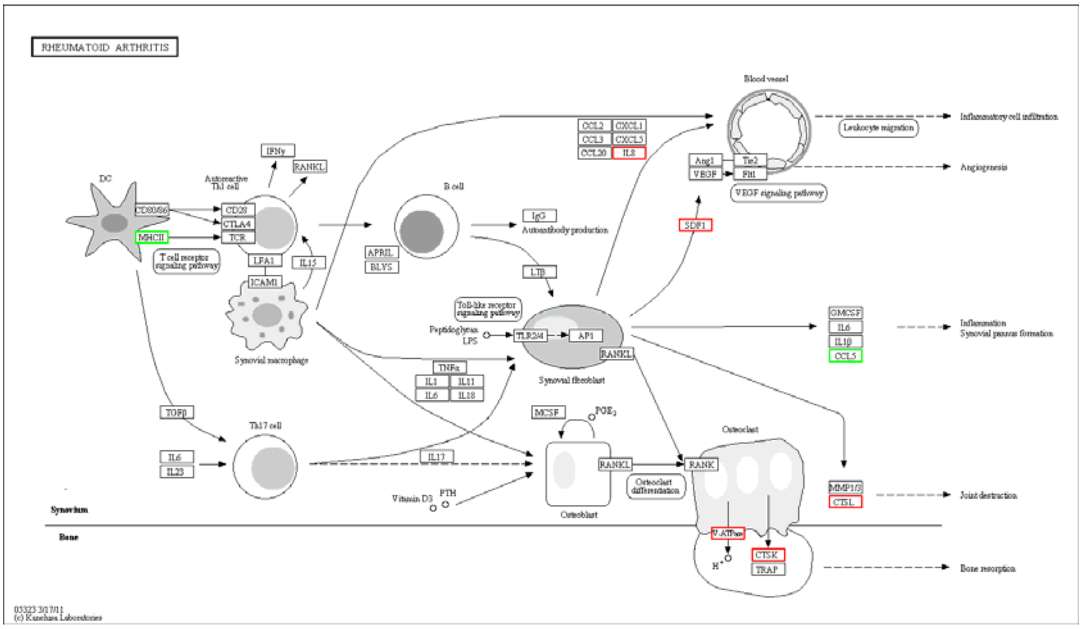

Figure S5.The significantly DEG-enriched KEGG rheumatoid arthritis pathway.

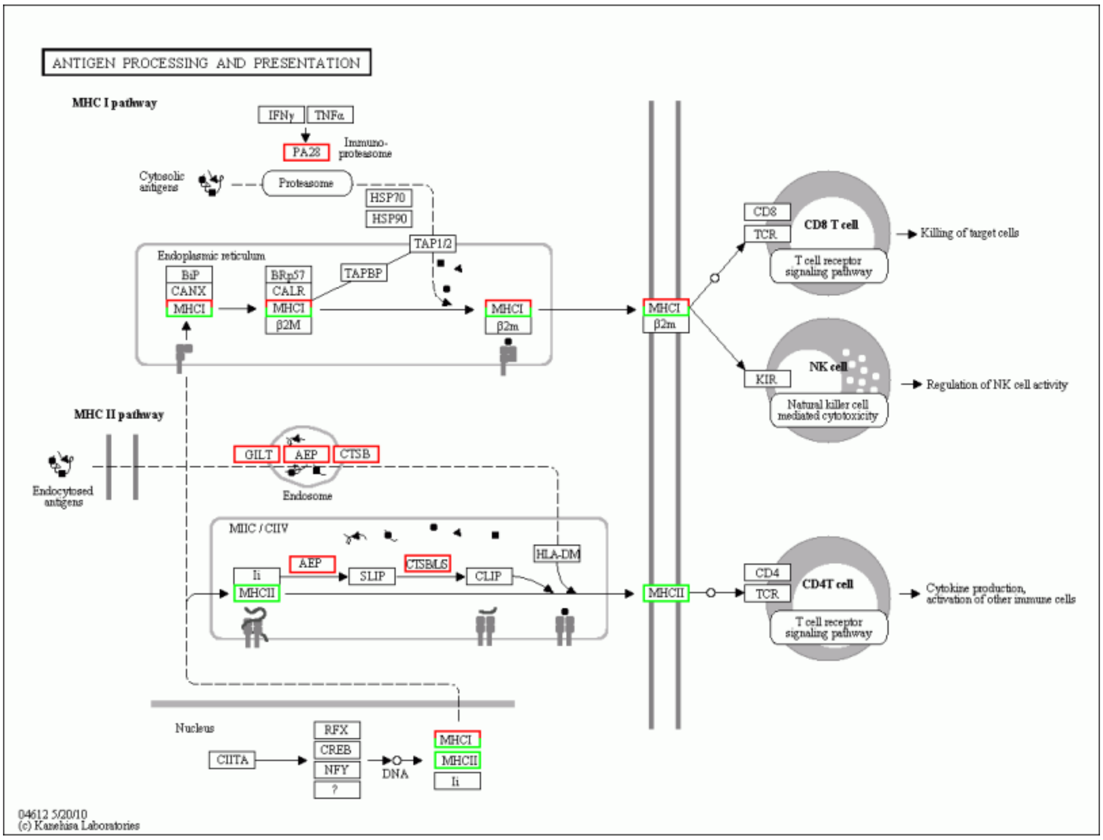

Figure S6. The significantly DEG-enriched KEGG antigen processing and presentation pathway.

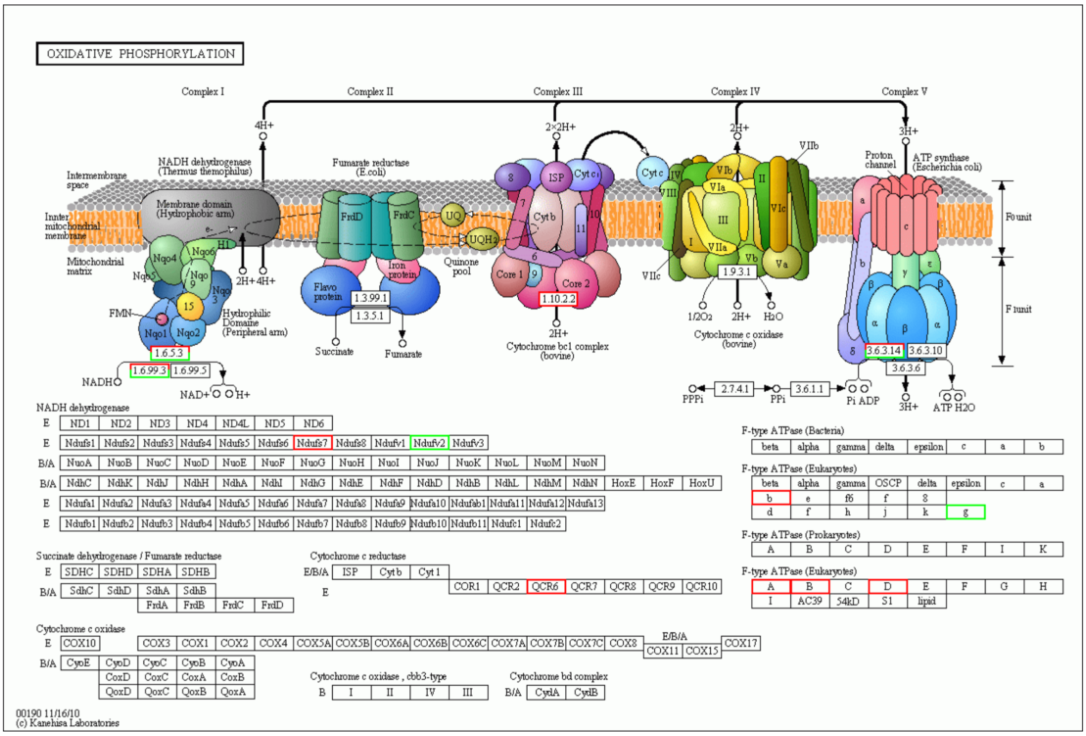

Figure S7. The significantly DEG-enriched KEGG oxidative phosphorylation pathway.

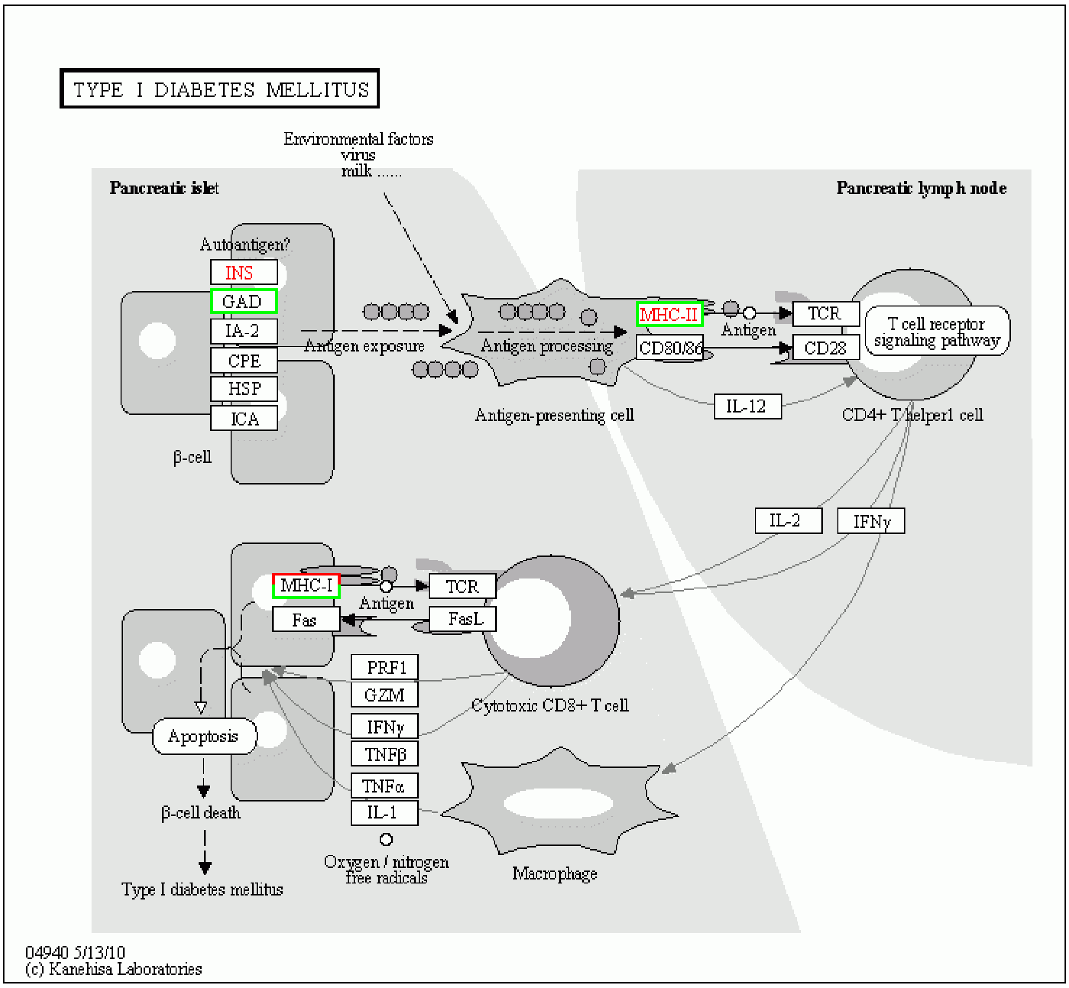

Figure S8. The significantly DEG-enriched KEGG type I diabetes mellitus pathway.

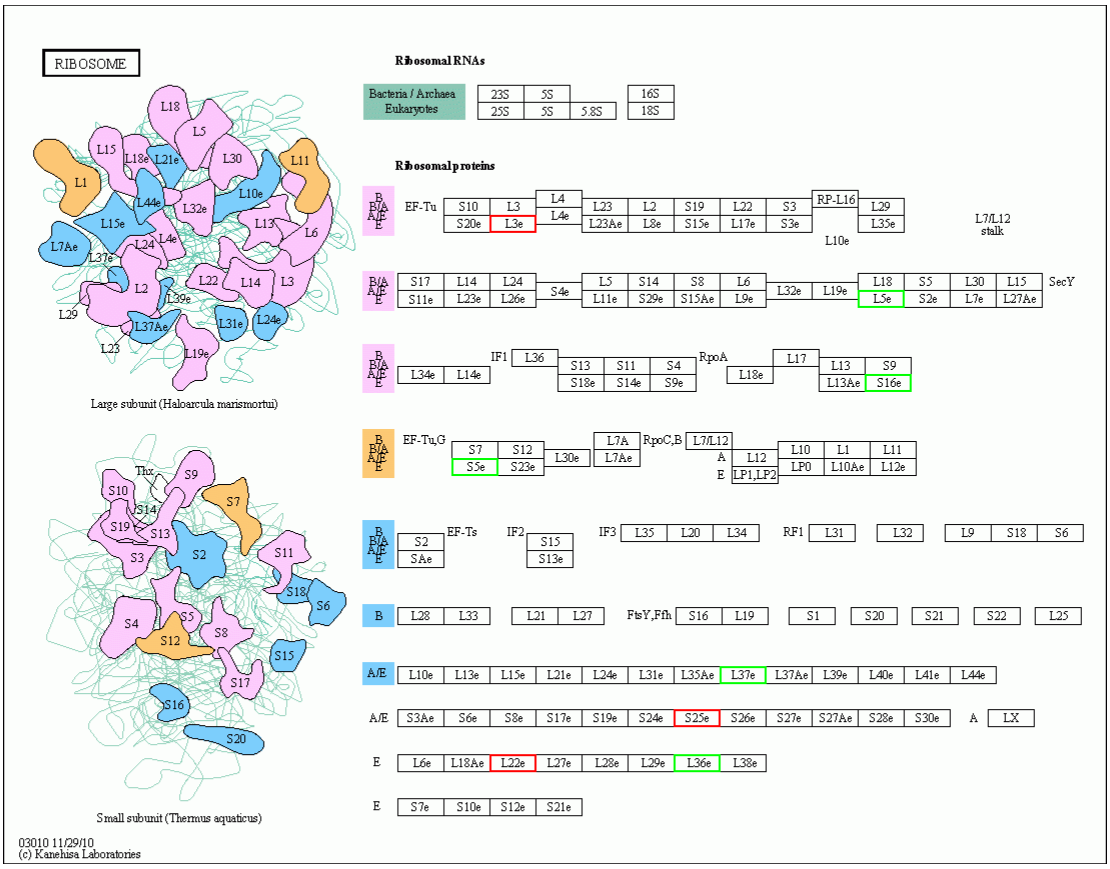

Figure S9. The significantly DEG-enriched KEGG ribosome pathway.
